# Long-Term Results of Hepatic Resection for Hepatolithiasis

**DOI:** 10.1155/1995/54872

**Published:** 1995

**Authors:** M. Sato, Y. Watanabe, S. Horiuchi, Y. Nakata, N. Sato, Y. Kashu, S. Kimura

**Affiliations:** Department of Surgery II Ehime University School of Medicine Ehime Japan

## Abstract

Long-term results of hepatic resection for hepatolithiasis in 34 patients having intrahepatic biliary strictures were studied. The left lateral and the right posterior segmental ducts were commonly and often simultaneously involved. Fourteen patients had multiple segmental involvement. Hepatic resection included left sided resection (*n*=27), right sided resection (*n*=6), and repeated bilateral resection (*n*=1). Seven patients had biliary tumors: 3 cholangiocarcinomas, 2 gall bladder cancers, cystadenocarcinoma, and dysplasia of intrahepatic ducts. Nineteen patients received bilioenteric anastomosis. Retained stones and recurrent stones developed in 3 and 4 patients, respectively. Twenty-six patients had no remaining symptoms; 2 died of operative complication or cholangiocarcinoma; 6 presented symptoms caused by retained stones (*n*=2), recurrent stones (*n*=2), bile stasis (*n*=1), or neuralgia (*n*=1). In 4 of the 6 patients, unrelieved posterior duct strictures caused the symptoms. With a mean follow-up period of 4.5 years, 30 patients are symptoms free, and 27 are stone free. In patients with right lobar or bilobar type, intra- and extrahepatic type, and confluence strictures, bilioenteric anastomosis is required. Hepatic resection is a rational treatment for hepatolithiasis, however, meticulous management of biliary tract abnormalities, particularly the posterior duct stricture, is mandatory.


*HPB Surgery*, 1995, Vol. 9, pp. 37–41
Reprints available directly from the publisher
Photocopying permitted by license only

© 1995 OPA (Overseas Publishers Association)
Amsterdam B.V. Published in The Netherlands
by Harwood Academic Publishers GmbH
Printed in Malaysia

### Long-Term Results of Hepatic Resection for Hepatolithiasis

M. SATO, Y. WATANABE, S. HORIUCHI, Y. NAKATA, N. SATO, Y. KASHU, and S. KIMURA

Department of Surgery II, Ehime University School of Medicine, Ehime, Japan

Long-term results of hepatic resection for hepatolithiasis in 34 patients having intrahepatic biliary strictures were studied. The left lateral and the right posterior segmental ducts were commonly and often simultaneously involved. Fourteen patients had multiple segmental involvement. Hepatic resection included left sided resection ($n$=27), right sided resection ($n$=6), and repeated bilateral resection ($n$=1). Seven patients had biliary tumors: 3 cholangiocarcinomas, 2 gall bladder cancers, 1 cystadenocarcinoma, and 1 dysplasia of intrahepatic ducts. Nineteen patients received bilioenteric anastomosis. Retained stones and recurrent stones developed in 3 and 4 patients, respectively. Twenty-six patients had no remaining symptoms; 2 died of operative complication or cholangiocarcinoma; 6 presented symptoms caused by retained stones ($n$=2), recurrent stones ($n$=2), bile stasis ($n$=1), or neuralgia ($n$=1). In 4 of the 6 patients, unrelieved posterior duct strictures caused the symptoms. With a mean follow-up period of 4.5 years, 30 patients are symptoms free, and 27 are stone free. In patients with right lobar or bilobar type, intra- and extrahepatic type, and confluence strictures, bilioenteric anastomosis is required. Hepatic resection is a rational treatment for hepatolithiasis, however, meticulous management of biliary tract abnormalities, particularly the posterior duct stricture, is mandatory.

KEY WORDS: Hepatolithiasis biliary stricture hepatic resection bilioenteric anastomosis choledochoscopy

### INTRODUCTION

The main goal of treatment for hepatolithiasis consists of complete removal of stones and elimination of bile stasis. The presence of intrahepatic biliary strictures[1–3] represents a clinical problem as to the management of this disease. Among various treatment modalities[4–8], hepatic resection can offer a chance of cure for patients with primary hepatolithiasis by eradicating stones, strictures, and diseased hepatic parenchyma. However, for complicated patients, appropriate procedures for biliary tract abnormalities together with hepatic resection are needed for successful treatment. This article summarizes our results of hepatic resection in the past 10 years in 34 patients with hepatolithiasis. We stress the importance of thorough retrieval and proper management of intrahepatic biliary abnormalities.

### PATIENTS AND METHODS

Between January 1985 and August 1994, 34 patients (13 men and 21 women) with hepatolithiasis having intrahepatic biliary stricture underwent elective hepatic resections. The patients' age ranged from 37 to 84 years with a mean of 60 years. All patients had varying degrees of cholangitis. Six patients presented with acute pancreatitis; 2 presented with a liver abscess. Seven patients had biliary tumors, including 3 cholangiocarcinomas, 2 gall bladder cancers, 1 hepatic cystadenocarcinoma, and 1 dysplasia of the intrahepatic ducts. Nineteen patients had undergone more than one previous biliary operation as follows: cholecystectomy ($n$=19), choledochotomy ($n$=11), choledochojejunostomy

*Address for correspondence*: Dr. Motomichi Sato, Department of Surgery II, Ehime University School of Medicine, Shigenobu, Onsen-gun, Ehime 791-02, Japan, Fax: 899/64-7491.

($n$=3), sphincteroplasty or endoscopic sphincterotomy of the ampulla of Vater ($n$=4), and drainage of liver abscess ($n$=2).

Before surgery, patients underwent a full radiological examination including sonography, computed tomography, endoscopic retrograde cholangiography, and/or percutaneous transhepatic cholangiography. All patients underwent cholangioscopy and/or cholangiography both during and after surgery. Stones were removed endoscopically using an electrohydraulic lithotriptor[5] or a basket catheter. Referring to the Japanese classification of hepatolithiasis[9], patients were classified as follows: bilobar type (type LR) in 9 patients, left lobar type (type L) in 19, and right lobar type (type R) in 6; intrahepatic type (type 1) in 14 patients and both intra-and extrahepatic type (type IE) in 20. Intrahepatic bile ducts were defined according to Healey' anatomy of the intrahepatic ducts[10] and abbreviated as follows: the hepatic duct (HD), the left lateral segmental duct (LD), the left median segmental duct (MS), the right anterior segmental duct (AS ), and the right posterior segmental duct (PS).

The indication for hepatic resection was when unextractable stones behind marked intrahepatic biliary strictures or marked hepatic atrophy were present. In cases having bilateral involvement, we resected the more damaged side of the liver, usually the left side. One-stage bilateral hepatic resection was avoided. Left lateral segmentectomy is the most common procedure among various types of hepatic resection (Table 1). One patient who received left lateral segmentectomy also underwent right posterior segmentectomy because of retained stones in the posterior ducts. In patients with bilobar involvement, dilated bile duct, or extrahepatic involvement, Roux-en-Y bilioenteric anastomoses were constructed for postoperative choledocoscopy (POC). As for the levels of bilioeneteric anastomoses, we used the common hepatic duct (hepatico-jejunostomy:HJ) in cases without remaining strictures, or bilateral hepatic ducts (hilar hepaticojejunostomy: hHJ) in cases with bilateral involvement, confluence strictures, or unrelieved strictures in the contralateral lobe. In hilar HJ, bilateral hepatic ducts were opened by a longitudinal incision beyond the strictures of the confluence up to the point where we could see the orifices of individual segmental ducts. Endoscopic management included postoperative biliary dilation in 2 patients, lithotomy in 14 patients (before surgery: 1, during surgery: 9, and after surgery: 6), and strictomy in one patient.

The precise locations of stones and strictures in individual intrahepatic ducts were determined by cholangiograms. The mean follow-up period was 4.5 years ranging from 0.5 to 9.5 years. The results of surgery were assessed in August 1994 and classified according to symptoms related to cholangitis after surgery as follows: good when they experienced no symptoms, fair when they had episodes of mild cholangitis which subsides at present, and poor when they are receiving special treatment for further cholangitis.

### RESULTS

Thirty-one patients had brown pigment stones, while the other 3 had cholesterol stones. Packed stones and segmental atrophy of the liver were found in 31 and 25 patients, respectively. Anomalies of biliary tracts included anomalous joining posterior ducts to the left hepatic duct in 4 patients, left-sided gall bladder in 1, and Situs inversus in 1. A total of 58 segmental ducts had stones, 36 of which were packed stones (Table 2). In 14 cases (41%), stones were located in two or more segments, including LS + PS in 7 cases, LS+MS in 3, AS+PS in 2, LS+MS+PS in 1, and all segments in 1. LS and PS were involved commonly and simultaneously.

**Table 1** Surgical procedures of hepatolithiasis

| Biliary tract management | no. of cases | Hepatic resection | no. of cases |
|---|---|---|---|
| Bilioenteric anastomosis | 19 | Left lateral segmentectomy | 22 |
| (hHJ:8, HJ:7 iCJ+HJ:3, right HJ:1) | | Left lobectomy | |
| Biliary drainage alone | 13 | Right posterior segmenectomy | 2 |
| Repair of biliary strictures | 5 | Right subsegmentectomy | 2 |
| (left HD:3, right HD:1, PS:1) | | Right lobectomy | 1 |
| Biliary dilation | 3 | Right anterior segmentectomy | 1 |
| (balloon:1, stenting:2) | | Repeated bilateral resection | 1 |
| Whipple's operation* | 1 | | |

hHJ: hilar hepaticojejunostomy, HJ: hepaticojejunostomy, iCJ: intrahepatic cholangiojejunostomy, HD: hepatic duct, and PS: right posterior segemental duct.

*Whipple's procedure was performed for carcinoma of the gall bladder.

Stones in MS and PS were detected during and after surgery in many cases. Operative sonography could disclose stones which choledochoscopy had failed to demonstrate during surgery. Biliary strictures mostly associated with stones at the orifices of each segmental duct were present in a total of 46 intrahepatic ducts. Half of the strictures were severe. Four strictures (PS in 3 cases and LS in 1 case) were not accompanied by stones. Eleven patients had multiple biliary strictures as follows: LS+PS in 6 patients, LS+HD in 2, LS+MS in 1, AS+PS in 1, and LS+PS+HD in 1. Extrahepatic biliary strictures were not present.

Eight patients had retained stones in the posterior ducts after surgery. All 8 patients were type IE; 7 of the 8 patients were bilobar type. Five of the 8 underwent complete stone clearance by POC (n=4) or repeated hepatic resection (n=1). POC failed in 4 patients who had unrelieved stricture of the posterior duct or right hepaticojejunostomy, which led to retained stones at discharge in 3 patients (Table 3). Figure 1 shows cholangiograms of a patient with multiple retained stones behind the unrelieved stricture of the right posterior duct which were detected by POC. The causes of retained stones after surgery included overlooking stones ($n$=5), remaining unresected part of the segment VII ($n$=1), insufficient stone removal by intraoperative choledochoscopy ($n$=1), and the presence of gall bladder cancer ($n$=1). Five patients who underwent complete stone clearance after surgery did not develop recurrent stones.

One patient who received left lateral segmentectomy for impacted stones behind the stricture in this segment had unrelieved mild stricture of the posterior duct which was not accompanied by stones (Table 3). She subsequently developed progressive stricture of both the posterior duct and the anterior duct. She presented cholangitis even without residual stones. She needed transhepatic insertion of an internal bypass catheter between the posterior duct and the left hepatic duct to minimize the bile stasis. She had an anomalous joining posterior duct to the left hepatic duct.

Recurrent stones were detected in 4 patients 3 to 7 years after hepatic resection (Table 3). Only one patient received biliary reconstruction at the time of hepatic resection. Three patients underwent complete stone clearance by either HJ or endoscopic sphincterotomy. Thereafter, one of the 3 patients developed anastomotic stenosis of the HJ and recurrent stones. This patient received percutaneous transhepatic choledochoscopic lithotomy and drainage, but she will need further surgery.

Excluding 3 patients (2 patients who died of surgery or recurrent cholangioma and one patient who suffers from liver cirrhosis associated with hepatitis C virus), the present conditions of 31 patients were good in 25 patients, fair in 4, and poor in 2. Six patients presented remaining symptoms after surgery due to retained stones ($n$=2), recurrent stones ($n$=2), bile stasis ($n$=1), and neuralgia ($n$=1). In 4 of the 6 patients, posterior duct stricture caused the symptoms. At present, 27 surviving patients are stone free; 30 patients are symptoms free.

### DISCUSSION

Since the affected part of the liver is gradually destroyed and replaced by fibrous tissue due to repeated cholangitis in hepatolithiasis, the segmental atrophy is a good candidate for hepatic resection[2,3,7]. High occurrence of cholangiocarcinoma in atrophic segments as in our series is another reason which justifies hepatic resection for this disease. As to the extent of hepatic resection, the resection line should be wide enough to remove the diseased part completely. Incomplete hepatic removal is more likely to occur in right sided hepatic

**Table 2** Locations of intrahepatic stones and strictures and the timing of endoscopic diagnosis

| Affected ducts | Intrahepatic stones (no. of cases) Total | Diagnostic timing pre- | during- | post-op | Biliary strictures (no. of cases) Total | severe |
|---|---|---|---|---|---|---|
| HD | 9 | 9 | 0 | 0 | 6 | 2 |
| LS | 27 | 27 | 0 | 0 | 24 | 15 |
| MS | 5 | 0 | 5 | 0 | 1 | 0 |
| AS | 4 | 3 | 1 | 0 | 1 | 1 |
| PS | 13 | 7 | 2 | 4 | 14 | 7 |
| Total | 58 | 46 | 8 | 4 | 46 | 25 |

HD: hepatic duct, LS: left lateral segmental duct, MS: left median segmental duct, AS: right anterior segmental duct, PS: right posterior segmental duct, and op.: operation.

The diagnosis of the intrahepatic stones or strictures was confirmed only when choledochoscopy and/or choledochography demonstrated their presence.

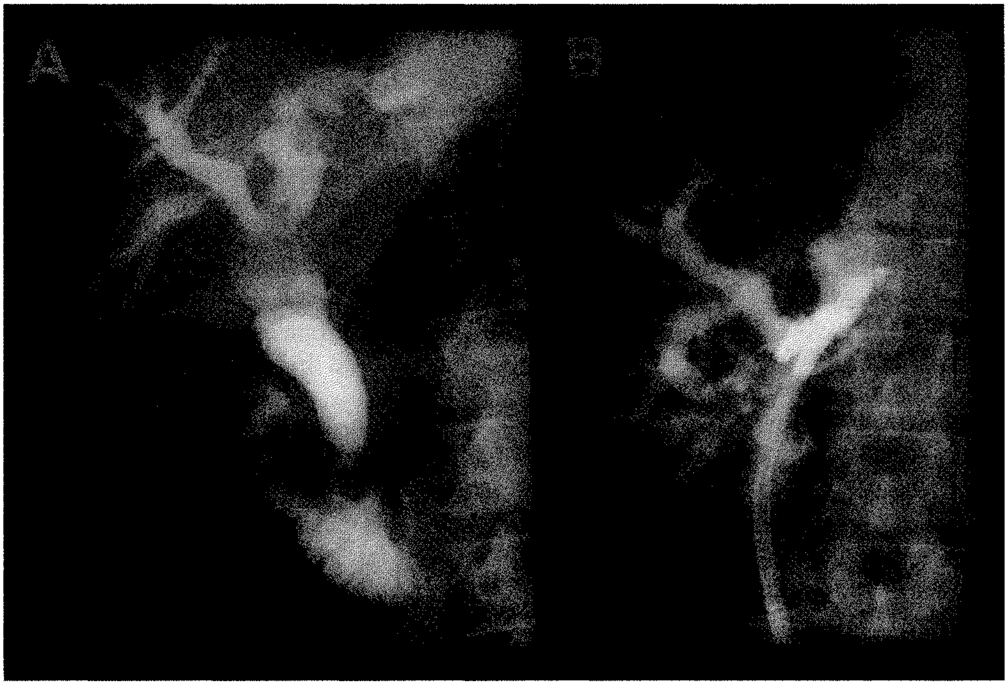

**Figure 1** Cholangiograms of a patient who had retained stones after surgery. A: preoperative endoscopic retrograde cholangiogram, B: postoperative endoscopic cholangiogram. Impacted stones in the right posterior segmental duct was not visualized on a preoperative cholangiogram, but the postoperative choledochoscopy disclosed the presence of retained stones and strictures in the posterior segment.

resection as in our series.

This study clarifies the features of intrahepatic biliary tract abnormalities. The result is better in left unilateral type patients than right or bilobar type patients. We should pay attention to the left median segment in case of left sided hepatic resection, since stones in the median segment are difficult to diagnose preoperatively. In cases having fibrosis in the median segment, or confluence stricture, left hepatic lobectomy is advocated rather than lateral segmentectomy to avoid re-

**Table 3** Restained stones, recurrent stones, and bile stasis after surgery

| Case | Disease | Initial | Location | | | At present | |
|---|---|---|---|---|---|---|---|
| | | | Stones | Biliary | | | |
| no. | type | procedure | (no.stones) | structure | Treatment | Symptoms | Stones |
| Retained stones | | | | | | | |
| 1 | R, IE | RPS, T | PS (several) | none | none | fair | present |
| 2 | LR, IE | LL, rHJ | PS (multiple) | PS, rHJ | POC | fair | present |
| 3 | LR, IE | LLS, T | PS (several) | PS | none | good | present |
| Recurrent stones | | | | | | | |
| 4 | L, IE | LLS, T | CBD (single) | none | EST | good | absent |
| 5 | R, I | RSS, T | CBD (multiple) | none | HJ | fair | absent |
| 6 | R, IE | RSS, T | CBD, all (multiple) | HJ | HJ -POC, PTBD | poor | present |
| 7 | R, I | RAS, hHJ | PS (single) | PS | none | good | present |
| Bile stasis | | | | | | | |
| 8 | L, I | LLS, T | none | PS, AS | biliary catheter | poor | absent |

L: left lobar type, R: right type, LR: both left and right type, I: intrahepatic type, IE: intra-and extrahepatic type, LLS: left lateral segmentectomy, LL: left lobectomy, RPS: right posterior segmentectomy, RSS: right sub segmentectomy, RAS: right anterior segmentectomy, HJ: hepaticojejunostomy, hHJ: hilar hepaticojejunostomy, rHJ: right HJ, T: choledochotomy and T-tube drainage, PS: right posterior segmental duct, AS: right anterior segmental duct, CBD: common bile duct, POC: postoperative choledochoscopy, and PTBD: percutaneous transhepatic biliary drainage.

tained stones in this segment. Meticulous retrieval and suitable management of the posterior segmental duct are key points for successful treatment. First, the posterior duct is the second most commonly affected duct, often simultaneously with the left lateral segmental duct. Second, stones and strictures in the posterior duct are likely to be overlooked by preoperative radiological investigations or even by intraoperative choledochoscopy. In our series, all retained stones following left sided hepatic resection remained in the posterior ducts. POC was successful only when there was no residual stricture of this duct. Third, the anomalous drainage pattern[11] and the sharp angulations of the posterior duct leads to the failure of choledocoscopy during surgery.

Only 3 patients had residual stones at discharge, but the other 5 had retained stones after surgery. Non-opacification of the affected ducts caused by biliary obstruction due to packed stones or strictures was the main cause of retained stones in most cases. All segmental ducts, particularly the posterior duct, should be demonstrated definitely with cholangioscopy and cholangiography using fluoroscopy during surgery and operative sonography. After surgery, we should perform POC in all cases to detect missed stones.

Patients with bilobar involvement, both extra-and intrahepatic involvement, and multiple biliary strictures are likely to develop retained stones after surgery. Patients having right lobar involvement, or receiving no biliary bypass are at high risk of recurrent stones. For these cases, bilioenteric anastomoses should be constructed as access routes for POC. In cases with bilateral involvement, multiple strictures, or confluence strictures, we advocate hilar HJ rather than HJ, because it relieves confluence strictures and provides direct visualization of all segmental ducts, and wide stomas. We believe that the hepatic hilum, including bilateral hepatic ducts and all orifices or segmental ducts, should be left unresected to avoid late anastomotic stenosis even in cases undergoing hemihepatectomy.

In conclusion, our results justify hepatic resection for hepatolithiasis with intrahepatic biliary strictures. Meticulous retrieval of non-opacified ducts is important to delineate all stones and strictures. Proper management of biliary tract abnormalities, particularly posterior duct stricture, is essential for successful treatment. In cases having posterior duct stricture, through management such as long-term dilatation[1,6,8], or staged bilateral hepatic resection is required to eliminate the pathophysiology.

### REFERENCES

1. Jeng, K.S., Yang, F.S., Ohta, I. and Chiang, H.J. (1990) Dilatation of intrahepatic biliary strictures in patients with hepatolithiasis. *Word J. Surg.*, **14**, 587–593.
2. Koga, A., Miyazaki, K., Ichimiya, H. and Nakayama, F. (1986) Choice of treatment for hepatolithiasis based on pathological findings. *World J. Surg.*, **8**, 36–40.
3. Fan, S.T., Choi, T.K., Lo, C.M., Mok, F.P.T., Lai, E.C.S. and Wong J. (1991) Treatment of hepatolithiasis: Improvement of result by a systemic approach. *Surgery*, **109**, 474–480.
4. Yamakawa, T. (1984) Percutaneous transhepatic stone extraction technique for management of retained biliary tract stones. In Intrahepatic calculi., edited by Okuda K., Nakayama F. and Wong J., pp. 253–268. New York: Alan R Liss Inc.
5. Yoshimoto, H., Ideka, S., Tanaka, M., Matsumoto, S. and Kuroda, Y. (1989) Choledochoscopic electrohydraulic lithotripsy and lithotomy for stones in the common bile duct, intrahepatic stones, and gall bladder. *Ann. Surg.*, **210**, 576–582.
6. Hutson, D.G., Russell, E., Schiff, E., Levi, J.J., Jeffers, L. and Zeppa R. (1984) Balloon dilation of biliary strictures through a choledochojejuno-cutaneous fistula. *Ann. Surg.*, **199**, 637–647.
7. Fan, S.T., Lai, E.C.S. and Wong, J. (1993) Hepatic resection for hepatolithiasis. *Arch. Surg.*, **128**, 1070–1074
8. Pitt, H.A., Venbrux, A.C., Coleman, J., Prescott, C.A., Johnson, M.S., Osterman, Jr. F.A., and Cameron, J.L. (1994) Intrahepatic stones. The transhepatic team approach. *Ann. Surg*, **219**, 527–537.
9. Nakayama, F., Furusawa, T., Nakama, T., *et al.* (1984) Clinical features and classification of hepatolithiasis. In Intrahepatic calculi., edited by Okuda K., Nakayama F., and Wong J., pp. 115–127, New York: Alan R Liss Inc.
10. Healey, Jr. J.E. and Schroy, P.C. (1953) Anatomy of the biliary ducts within the human liver. *Arch. Surg.*, **66**, 599 616.
11. Cullingford, G., Davidson, B., Dooley, J. and Habib N. (1991) Hepatolithiasis associated with anomalous biliary anatomy and a vascular compression. *HPB Surgery* **3**, 129–137.

